# Adjusting kinematics and kinetics in a feedback-controlled toe walking model

**DOI:** 10.1186/1743-0003-9-60

**Published:** 2012-08-25

**Authors:** Andrej Olenšek, Zlatko Matjačić

**Affiliations:** 1, University Rehabilitation Institute, Republic of Slovenia, Linhartova 51, 1000 Ljubljana, Slovenia

**Keywords:** Toe walking, Two-level control strategy, Control parameters, Gait analysis, Gait kinematics, Gait kinetics

## Abstract

**Background:**

In clinical gait assessment, the correct interpretation of gait kinematics and kinetics has a decisive impact on the success of the therapeutic programme. Due to the vast amount of information from which primary anomalies should be identified and separated from secondary compensatory changes, as well as the biomechanical complexity and redundancy of the human locomotion system, this task is considerably challenging and requires the attention of an experienced interdisciplinary team of experts. The ongoing research in the field of biomechanics suggests that mathematical modeling may facilitate this task. This paper explores the possibility of generating a family of toe walking gait patterns by systematically changing selected parameters of a feedback-controlled model.

**Methods:**

From the selected clinical case of toe walking we identified typical toe walking characteristics and encoded them as a set of gait-oriented control objectives to be achieved in a feedback-controlled walking model. They were defined as fourth order polynomials and imposed via feedback control at the within-step control level. At the between-step control level, stance leg lengthening velocity at the end of the single support phase was adaptively adjusted after each step so as to facilitate gait velocity control. Each time the gait velocity settled at the desired value, selected intra-step gait characteristics were modified by adjusting the polynomials so as to mimic the effect of a typical therapeutical intervention - inhibitory casting.

**Results:**

By systematically adjusting the set of control parameters we were able to generate a family of gait kinematic and kinetic patterns that exhibit similar principal toe walking characteristics, as they were recorded by means of an instrumented gait analysis system in the selected clinical case of toe walking. We further acknowledge that they to some extent follow similar improvement tendencies as those which one can identify in gait kinematics and kinetics in the selected clinical case after inhibitory casting.

**Conclusions:**

The proposed walking model that is based on a two-level control strategy has the ability to generate different gait kinematics and kinetics when the set of control parameters that define walking premises change. Such a framework does not have only educational value, but may also prove to have practical implications in pathological gait diagnostics and treatment.

## Background

By providing detailed insight into the physiology of human walking, extensive clinical application of instrumented gait analysis has considerably deepened our understanding of human locomotion mechanisms and has significantly improved the accuracy and reliability of pathological gait assessment [1-4]. On the other hand, being faced with a vast amount of information that required proper interpretation, clinicians, therapists and biomechanists were compelled to combine efforts to properly process and interpret the available walking information in order to reach the decision about the most promising therapeutical intervention. This task is even more challenging if we consider that gait pattern is often changed due to a combined effect of more than one impaired muscle functionalities and/or bone deformities rather than due to an isolated gait anomaly [3,4]. Despite continuous efforts, to date no general or standardized methodical approach has been adopted that would enable straightforward data interpretation and pathological gait diagnostics, let alone provide a reliable forecast about the most likely outcome of individual therapeutical intervention.

However, the ongoing research in the field of biomechanics suggests that a vast amount of unused potential may be available in biomechanical modeling and simulation. Mathematical modeling of the human gait departs from the actual biomechanical system and describes the properties of the human musculo-skeletal system with corresponding mathematical models. Such decomposition grants the user unlimited access to all of the model parameters. In terms of human movement analysis and pathological gait diagnostics and treatment, the user would strive to identify a particular anomaly by determining the model parameters that induce the underlying pathology. In the subsequent decision making process the user would ideally want to estimate the most likely outcome after selected therapeutical intervention by adjusting these parameters in such a way as to encode the physiological changes due to particular intervention. The efficiency of such an approach inevitably depends on the ability authentically to model the human locomotion apparatus and physiological processes and also on how the motion itself is being generated. There are two mainstreams being followed when generating motion, and they have proven to have great potential for practical applicability in human gait interpretation and analyses: optimization-based modeling [5-19] and control-based modeling [20-27].

Optimization-based models generate joint motions and joint forces by optimizing human related performance criteria subject to physical constraints [5-16,19]. The key issue of such an approach is the selection of appropriate objective criteria and corresponding constraints [6]. If prerecorded motion data are available, the objective criteria reflect the actual physiology of human motion. They offer realistic and arbitrarily detailed modeling of the human locomotion system, which has already proven invaluable in interpretations of normal as well as pathological human gait [14,16-18]. However, in motion prediction it is doubtful whether if new optimization criteria can be adequately formulated, since they should relate to the motion which is actually the subject of prediction. Also, when formulating an optimization problem one must bear in mind the required computation time. In order to reach an optimal solution on a large scale musculo-skeletal model, the amount of required computational effort is enormous. To some extent computational demands may be mitigated by applying control-based models.

Control-based models utilize various control algorithms to calculate joint actuations that drive the biped to follow predefined trajectories [20-24,28,29]. They are often embedded in the optimization-based walking models. First, feedback control is used to obtain joint actuations that impose the desired kinematics obtained by means of motion capture system, and afterwards static optimization techniques compute the muscle excitation [24-26]. Since this approach can produce high detailed motion on the level of muscle forces, it seems to be tailored for the interpretation of patient-specific pathological gait and muscle tendon force prediction. On the other hand, the necessity for a priori walking demands does interfere with the possibility of motion prediction. Since a priori walking demands are a prerequisite in the control-based approach, the possibility of generating new movements that would reflect structural or control changes is considerably limited. It has been suggested, though, that an adaptive feedback control may be a promising approach. In [27] we proposed a two-level adaptive feedback control strategy that can generate toe walking kinematics and kinetics in a simple planar biped walking model that to some extent corresponds to principal walking characteristics as observed in clinical cases of toe walking. The method is based on defining a set of general walking premises rather than prescribing the actual joint angles, which forms a framework where joint angles do not depend solely on angle references but are free to develop within a set of desired walking premises.

In this paper we explore the possibility of developing a family of toe walking gait kinematic and kinetic patterns with distinguished toe walking characteristics by first parametrizing the desired walking premises and later introducing these parameters as gradually changing control objectives to the feedback-controlled toe walking model. We envision, that in interpretations of human gait pattern, the imposition of such changes may encode constrained walking premises due to an underlying primary anomaly, while in prediction of the therapy outcome they may encode expected walking premises due to selected therapeutical intervention, whereas the evaluation of resulting gait kinematics and kinetics at the end would identify the overall success of the selected therapeutical intervention.

## Methods

The following sections present a biped walking model and corresponding two-level control strategy in a condensed form. A detailed overview is available in [27].

### Biped walking model

**Figure 1:**
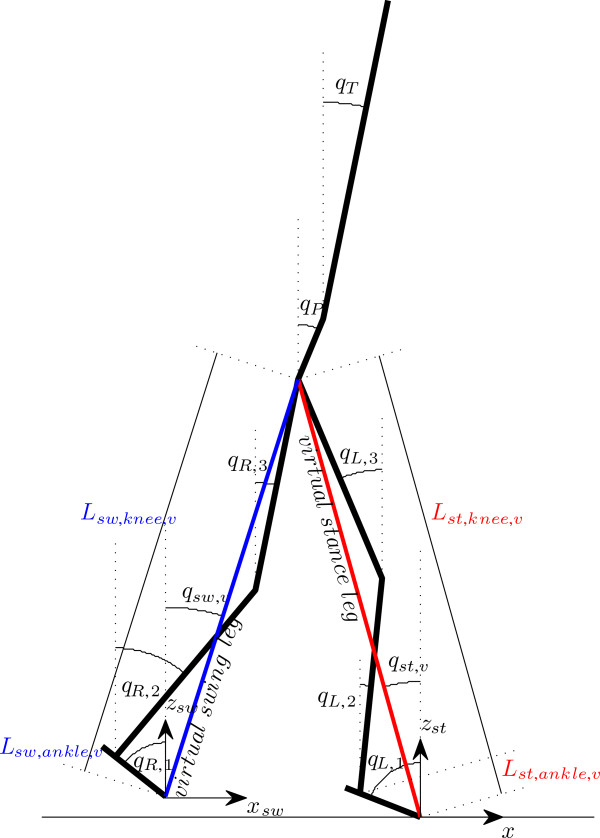
Schematic representation of the biped walking model.

The model considered is a planar biped walking model with eight body segments. Thigh, shank and foot segments are connected at hip, knee and ankle joints respectively, and carry the pelvis and torso segments (Figure 1). We consider a step to be a movement of the biped walking model between the contact of one leg and the successive contact of the opposite leg. Each step is divided into phases of double support and single support. Compared to the double support phase, when both legs are in contact with the ground, in the single support phase only the stance leg remains in contact with the ground and the opposite leg advances towards the point of next contact. Two consecutive steps constitute a complete gait cycle. By following a Lagrange formulation of the constrained system, the equations of motion in single support and double support can be formed as:

(1) $$\begin{array}{c} \begin{array}{c} M\ddot{q}+C\left(q,\dot{q}\right)\dot{q}+G(q)=\mathrm{Bu}+\Gamma_{\kappa }^{T}\lambda_{\kappa }\\ \;\;\Gamma_{\kappa }\dot{q}=\frac{\partial \Psi_{\kappa }}{\mathrm{\partial q}}\dot{q}=0 \end{array}\;\left|\kappa =\left\{\begin{array}{c} \mathrm{ss},\;\;\;\text{single support phase}\\ \mathrm{ds},\;\;\text{double support phase} \end{array}\right)\right) \end{array}$$

and written in the state space form as:

(2) $$\begin{array}{c} {\dot{x}}_{\kappa }=f_{\kappa }(x_{\kappa })+g_{\kappa }(x_{\kappa })\cdot u\;\left|\kappa =\left\{\begin{array}{c} \mathrm{ss},\;\;\;\text{single support phase}\\ \mathrm{ds},\;\;\text{double support phase} \end{array}\right)\right) \end{array}$$

Transition between single support and double support is referred to as the contact phase and is associated with the swing leg touching the ground. Likewise, transition between double support and single support is referred to as the take-off phase and is associated with the trailing leg lifting off the ground. Both transition phases are assumed to be instantaneous. The following set of algebraic equations describes both transition phases:

(3) $$\begin{array}{c} \begin{array}{c} \left[\begin{array}{c} M\;\;-\Gamma_{\varepsilon }^{T}\\ \Gamma_{\varepsilon }\;\;\;0 \end{array}\;\right]\cdot \left[\begin{array}{c} {\dot{q}}^{+}\\ F_{\varepsilon } \end{array}\;\right]=\left[\begin{array}{c} M{\dot{q}}^{-}\\ 0 \end{array}\;\right] \end{array}\;\left|\varepsilon =\left\{\begin{array}{c} \;\;c,\;\;\;\text{contact phase}\\ \mathrm{top},\;\;\text{take-off phase} \end{array}\right)\right) \end{array}$$

In (1) and (3) *q* is a set of configuration coordinates with superscript ^+ ^denoting the set of configuration variables just after the transition, and superscript ^−^ denoting the set of configuration variables just before the transition; *u* denotes the set of joint moments, *M* is the inertia matrix, *C* is the matrix of centripetal and Coriolis terms, *G* is the gravity vector, *Γ* accounts for constraints and *λ* is a set of negative ground reaction forces.

The control strategy applied in this toe walking model is a two level control strategy that on a lower, within-step control level in support phases imposes trajectory tracking via feedback control, and on the higher, between-step control level adaptively adjusts forward propulsion by changing virtual stance leg lengthening velocity at the end of the single support after each step so as to achieve stable gait at the desired gait velocity. In single and double support, general walking characteristics are encoded as a set of holonomic constraints and as outputs imposed on the toe walking model via feedback control. Observations of human walking in the single support phase suggest that humans move roughly symmetrically, the swing leg is lifted off the ground to assure sufficient foot clearance when advancing towards the point of new contact, vertical hip movement is minimized to prevent excessive energy consumption, whereas the pelvis as well as torso segments display slight oscillatory movement about the selected position close to the vertical. While this set of demands is sufficient for generating stable walking of the model, two additional demands are needed to calculate the two remaining joint moments. Since the aim of the model is to investigate changes in toe walking kinematics and kinetics subject to variations in control parameter values, these additional demands have been chosen to control the extent of ankle plantar flexion, which in toe walking is the main pathological characteristic. For this reason we introduced the virtual stance/swing leg (*L*_*st*,*v*_ and *L*_*sw*,*v*_ respectively), being the distance between the tip of stance/swing foot and the hip joint. The virtual stance/swing leg is further divided into the ankle component of the virtual stance/swing leg (*L*_*st*,*ankle*_ and *L*_*sw*,*ankle*_, respectively) and the knee component of the virtual stance/swing leg (*L*_*st*,*knee*_ and *L*_*sw*,*knee*_, respectively) (Figure 1). By selecting ankle components of both virtual legs as the remaining control objectives, general walking observations in single support can be incorporated in the following output vector:

(4) $$y_{\mathrm{ss}}\;=\;h_{\mathrm{ss}}\;=\;\left[\begin{array}{c} \;\;\;\;\;\;q_{\mathrm{st},v}+q_{\mathrm{sw},v}-(q_{\mathrm{st},v}+q_{\mathrm{sw},v})\cdot w_{\mathrm{ss},1}\\ z_{\mathrm{sw}}-\frac{L_{\mathrm{leg},\mathrm{nominal}}}{k_{1}}(q_{\mathrm{st},v,d}\cdot w_{\mathrm{ss},2}+q_{\mathrm{st},v}{\left|\right)}_{t=T_{\mathrm{ss},\mathrm{start}}}\cdot w_{\mathrm{ss},3}-q_{\mathrm{st},d})\\ \;L_{\mathrm{st},v}-L_{\mathrm{st},v,d}(q_{\mathrm{st},v})\\ \;q_{P}-q_{P,d}(q_{\mathrm{st},v})\\ \;q_{T}-q_{T,d}(q_{\mathrm{st},v})\\ \;L_{\mathrm{st},\mathrm{ankle}}-L_{\mathrm{st},\mathrm{ankle},d}(q_{\mathrm{st},v})\\ \;L_{\mathrm{sw},\mathrm{ankle}}-L_{\mathrm{sw},\mathrm{ankle},d}(q_{\mathrm{st},v}) \end{array}\right]$$

When zeroing out the *y*_*ss*_via feedback control, *w*_*ss*,1_ ensures exponential reduction of the asymmetry immediately after lifting the swing leg off the ground. Furthermore, controlling the foot clearance on one hand prescribes sufficient foot clearance to prevent the swing leg from hitting the ground while advancing, but also defines when the swing leg should touch the ground, thus ending the single support phase. Namely, assuming that the symmetry is being imposed via feedback control at the end of single support directly implies that the swing leg should touch the ground and terminate the single support phase exactly when

(5) $$q_{\mathrm{st},v}{|}_{t=T_{\mathrm{ss},\mathrm{end}}}=q_{\mathrm{st},v,d}$$

In (5) *q*_*st*,*v*,*d*_ denotes the desired virtual stance leg angle at the end of the single support phase and is related to desired gait velocity *v*_*gait*,*d*_, desired cadence *cad*_*gait*,*d*_ and desired step length *L*_*step*,*d*_ as

(6) $$\begin{array}{lll} L_{\mathrm{step},d}=\frac{2\cdot v_{\mathrm{gait},d}}{\mathrm{ca}d_{\mathrm{gait},d}} & =x_{\mathrm{st}}{|}_{t=T_{\mathrm{ss},\mathrm{start}}}-x_{\mathrm{sw}}{|}_{t=T_{\mathrm{ss},\mathrm{start}}}\; & \;\\ & \;+2\cdot L_{\mathrm{leg},\mathrm{nominal}}\cdot \sin(q_{\mathrm{st},v,d})\; \end{array}$$

**Figure 2:**
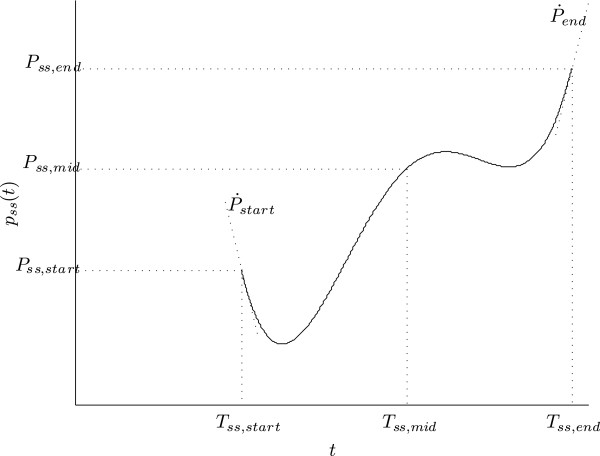
**Polynomial representation of the control objective.** Five parameters are needed for unique definition of the forth order polynomial.

Nominal length of the virtual leg *L*_*leg*,*nominal*_ and *k*_1_ determine how high the swing leg is lifted while advancing, whereas *w*_*ss*,2_ and *w*_*ss*,3_ assure smooth exponential transition. The remaining *L*_*st*,*v*,*d*_(*q*_*st*,*v*_), *q*_*P*,*d*_(*q*_*st*,*v*_), *q*_*T*,*d*_(*q*_*st*,*v*_), *L*_*st*,*ankle*,*d*_(*q*_*st*,*v*_) and *L*_*sw*,*ankle*,*d*_(*q*_*st*,*v*_) define the desired virtual stance leg length, desired pelvic movement, desired torso movement and desired lengths of ankle components of stance and swing leg respectively. They are all defined as fourth order polynomials. As shown in Figure 2, five parameters suffice to completely define the fourth order polynomial. The initial position and initial velocity for any of the five polynomials are determined according to the state of the model in the preceding take-off phase to assure position and velocity continuity, whereas the remaining three parameters may be user-defined to impose the desired gait characteristics. Five corresponding parameters for each of the selected reference trajectories are presented in Table 1.

**Table 1:** List of parameters defining fourth order polynomial representation in five selected reference trajectories

***p*_*ss*_(*t*)**	***L*_*st*,*v*,*d*_(*q*_*st*,*v*_)**	***q*_*P*_(*q*_*st*,*v*_)**	***q*_*T*_(*q*_*st*,*v*_)**	***L*_*st*,*ankle*,*d*_(*q*_*st*,*v*_)**	***L*_*sw*,*ankle*,*d*_(*q*_*st*,*v*_)**	
*P*_*ss*,*start*_	*L*_*st*,*v*,*start*_	*q*_*P*,*start*_	*q*_*T*,*start*_	*L*_*st*,*ankle*,*start*_	*L*_*sw*,*ankle*,*start*_	position continuity
*P*_*ss*,*mid*_	*L*_*st*,*v*,*mid*_	*q*_*P*,*mid*_	*q*_*T*,*mid*_	*L*_*st*,*ankle*,*mid*_	*L*_*sw*,*ankle*,*mid*_	user defined
*P*_*ss*,*end*_	*L*_*st*,*v*,*end*_	*q*_*P*,*end*_	*q*_*T*,*end*_	*L*_*st*,*ankle*,*end*_	*L*_*sw*,*ankle*,*end*_	user defined
${\dot{P}}_{\mathrm{ss},\mathrm{start}}$	${\dot{L}}_{\mathrm{st},v,\mathrm{start}}$	${\dot{q}}_{P,\mathrm{start}}$	${\dot{q}}_{T,\mathrm{start}}$	${\dot{L}}_{\mathrm{st},\mathrm{ankle},\mathrm{start}}$	${\dot{L}}_{\mathrm{sw},\mathrm{ankle},\mathrm{start}}$	velocity continuity
${\dot{P}}_{\mathrm{ss},\mathrm{end}}$	${\dot{L}}_{\mathrm{st},v,\mathrm{end}}$	${\dot{q}}_{P,\mathrm{end}}$	${\dot{q}}_{T,\mathrm{end}}$	${\dot{L}}_{\mathrm{st},\mathrm{ankle},\mathrm{end}}$	${\dot{L}}_{\mathrm{sw},\mathrm{ankle},\mathrm{end}}$	user defined

Due to the fact that both legs are in contact with the ground in double support, the available space of motions in double support is decreased by one degree of freedom as compared to single support. Therefore only six linearly independent output functions can be selected to describe the motion of the toe walking model in double support. Enforcing symmetry in the double support phase would interfere with forward progression and prevent proper weight transfer. Instead, the model must take into account small asymmetrical movement until lifting the trailing leg off the ground. Similarly, since both legs remain in contact with the ground throughout the double support, foot clearance is suspended from the set of output functions. Since the duration of double support is considerably smaller than the duration of single support, the expected range of motion in double support is considerably smaller. For this reason we selected the reference trajectories in double support as slow exponential functions (via *w*_*ds*,2_, *w*_*ds*,3_, *w*_*ds*,4_, *w*_*ds*,5_ and *w*_*ds*,6_), which yields the following output vector in double support:

(7) $$y_{\mathrm{ds}}=h_{\mathrm{ds}}=\left[\begin{array}{c} q_{\mathrm{st},v}+q_{\mathrm{sw},v}-(q_{\mathrm{st},v}+q_{\mathrm{sw},v}){|}_{t=T_{\mathrm{ds},\mathrm{start}}}\cdot w_{\mathrm{ds},1}\\ L_{\mathrm{st},v}-L_{\mathrm{st},v,\mathrm{end}}\cdot w_{\mathrm{ds},2}\\ q_{P}-q_{P,\mathrm{end}}\cdot w_{\mathrm{ds},3}\\ q_{T}-q_{T,\mathrm{end}}\cdot w_{\mathrm{ds},4}\\ L_{\mathrm{st},\mathrm{ankle}}-L_{\mathrm{st},\mathrm{ankle},\mathrm{end}}\cdot w_{\mathrm{ds},5}\\ L_{\mathrm{sw},\mathrm{ankle}}-L_{\mathrm{sw},\mathrm{ankle},\mathrm{end}}\cdot w_{\mathrm{ds},6} \end{array}\right]$$

After defining the output vectors in single and double support, the control objective is to drive the outputs *y*_*ss*_ and *y*_*ds*_ to zero. By following the standard Lie derivative notation the following feedback is applied in single support:

(8) $$u_{\mathrm{ss}}=-{\left(L_{g_{\mathrm{ss}}}L_{f_{\mathrm{ss}}}h_{\mathrm{ss}}\right)}^{-1}(L_{f_{\mathrm{ss}}}^{2}h_{\mathrm{ss}}+K_{D,\mathrm{ss}}L_{f_{\mathrm{ss}}}h_{\mathrm{ss}}+K_{P,\mathrm{ss}}h_{\mathrm{ss}})$$

Likewise, the following set of inputs will impose the desired movement in double support:

(9) $$u_{\mathrm{ds}}=-{\left(L_{g_{\mathrm{ds}}}L_{f_{\mathrm{ds}}}h_{\mathrm{ds}}\right)}^{+}(L_{f_{\mathrm{ds}}}^{2}h_{\mathrm{ds}}+K_{D,\mathrm{ds}}L_{f_{\mathrm{ds}}}h_{\mathrm{ds}}+K_{P,\mathrm{ds}}h_{\mathrm{ds}})$$

In (8) and (9), *L*_*g*_*L*_*f*_*h* denotes the decoupling matrix, *K*_*P*_ and *K*_*D*_ are positive definite gain matrices whereas the superscript ^+ ^indicates Moore-Penrose inverse.

**Figure 3:**
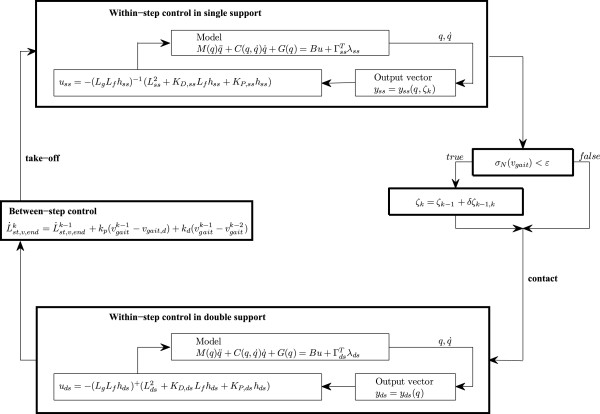
Schematic diagram of the two level control strategy.

Before starting the next single support phase, the higher between-step control level adaptively adjusts the virtual stance leg lengthening/shortening velocity at the end of single support ${\dot{L}}_{\mathrm{st},v,\mathrm{end}}$ according to gait velocity in the preceding step $v_{\mathrm{gait}}^{k-1}$. If the model walks slower than desired $v_{\mathrm{gait}}^{k-1}<v_{\mathrm{gait},d}$, then the model should accelerate, whereas if the model walks faster than desired $v_{\mathrm{gait}}^{k-1}>v_{\mathrm{gait},d}$, then the model should slow down. Preliminary simulation experiments [27] demonstrated that this is possible by increasing/decreasing push-off through subtle adjustments of ${\dot{L}}_{\mathrm{st},v,\mathrm{end}}$. This is encoded in the following between-step control algorithm:

(10) $${\dot{L}}_{\mathrm{st},v,\mathrm{end}}^{k}=L_{\mathrm{st},v,\mathrm{end}}^{k-1}+k_{p}(v_{\mathrm{gait}}^{k-1}-v_{\mathrm{gait},d})+k_{d}(v_{\mathrm{gait}}^{k-1}-v_{\mathrm{gait}}^{k-2})$$

where*k*_*P*_ and *k*_*D*_denote positive gains. Complete mechanism of the two-level feedback control strategy is illustrated in Figure 3.

We used Matlab and Matlab Simulink for development of the biped walking model and control strategy as well as for performing the simulation experiment.

### Generating different gait kinematics and kinetics

**Figure 4:**
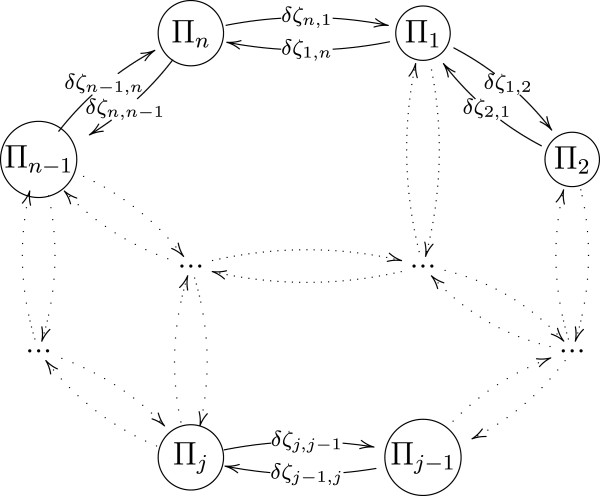
**Transitions between stable states π**_i_. After settling at desired gait velocity, changing the set of control objectives *ζ*will result in settling at new stable state with modified gait kinematics and kinetics.

It has been experimentally verified in [27] that adaptively adjusting ${\dot{L}}_{\mathrm{st},v,\mathrm{end}}$ after each step so as to enable gait velocity control significantly improves biped model robustness and stability. In our opinion this is the essential condition that would assure stability even if the values of selected control parameters were modified by the user with the intention of generating desired gait pattern characteristics. According to Table 1 fourteen control parameters remain available to define specific toe walking characteristics. Let 

 (11)

be the set of selected control parameters and let

(12) $$\begin{array}{lll} \pi & =\left\{\left(q_{\kappa }^{T},{\dot{q}}_{\kappa }^{T})\in \mathrm{TQ}\;|h_{\kappa }(q\right)\right)\; & \;\\ & =0,L_{f}h_{\kappa }(q)=0\;\left|\kappa =\left\{\begin{array}{c} \mathrm{ss},\;\;\;\text{single support phase}\\ \mathrm{ds},\;\;\text{double support phase} \end{array}\right)\right)\; \end{array}$$

denote gait kinematics and kinetics in double support and succeeding single support (i.e. in one step) after settling at the preselected gait velocity. Assuming that the toe walking model settles at the desired preselected gait velocity and that *y*_*ss*_ and *y*_*ds*_zero out after being imposed onto the model via feedback control, then each *π*_*j*_ may be considered as a stable state of the toe walking model that is uniquely identified with the set of control parameters *ζ*_*j*_. By changing the set of control parameters

(13) $$\zeta_{j}=\zeta_{j-1}+\delta \zeta_{j-1,j}$$

the model will develop new stable gait kinematics and kinetics *π*_*j*−1_→*π*_*j*_ in a finite number of steps *l* only if *δζ*_*j*−1,*j*_ is sufficiently small to prevent model destabilization. Since *ζ*_*j*_ uniquely defines the corresponding *π*_*j*_, in general there exists more than one sequence of *δζ*_*i*_*i* = *j*…*r*, that will eventually result in developing the same toe walking gait kinematics and kinetics. Likewise, by selecting such a sequence of *δζ*_*i*_*i* = *j*…*r* that *ζ*_*j*_ =*ζ*_*r*_, the toe walking model will return to the same stable state, hence *π*_*j*_ =*π*_*r*_. The concept of changing gait kinematics and kinetics by adjusting the set of control parameters is illustrated in Figure 4.

### Clinical case of toe walking

**Figure 5:**
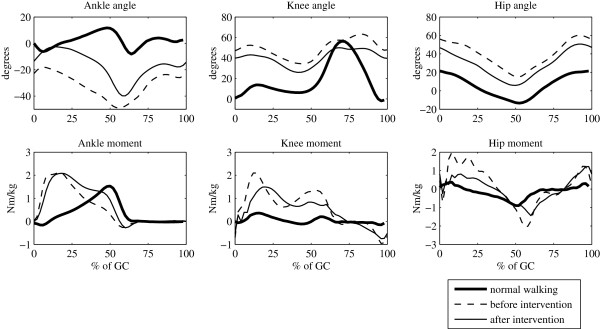
Clinical example of toe walking.

The aim of this study was to investigate through simulation experiment whether a sequence of*ζ*_*i*_ can be selected in such a way that the resulting family of simulated toe walking gait patterns would qualitatively follow kinematic and kinetic changes due to typical intervention in clinical practice. Considering that the small number of DOFs confines the feasible range of movements to a significantly smaller space than typically available in humans, we find incorporation of only principal toe walking characteristics in our toe walking model rather than exact reconstruction of selected gait kinematics and kinetics to be a rational compromise. For this reason we followed a typical clinical case of toe walking gait pattern as recorded in an eleven-year-old male subject before and after serial inhibitory casting (Figure 5). The subject was diagnosed with cerebral palsy, spastic diparesis, with excessive equinus gait. Before serial inhibitory casting he was three times treated with botulinum toxin due to excessive plantar flexor spasticity and once underwent serial inhibitory casting.

We used a standardized methodology for recording the subject’s gait kinematics and kinetics. 3D movements of IR reflective markers that were placed over palpable anatomical landmarks were recorded with the Vicon Mx motion capture system and were used in subsequent joint angle calculations. Simultaneously, we used two AMTI force plates that were positioned in the middle of the walkway to record ground reaction forces for subsequent joint moments calculations. A representative gait pattern was obtained by averaging gait kinematics and kinetics for both legs in at least three valid strides. A stride was considered to be valid if the same leg landed within the boundaries of one of the two force plates.

The recorded toe walking gait pattern allows us to observe the principal characteristics of toe walking. Compared to normal walking, the toe walking gait displays immediate movement towards ankle dorsal flexion after contact, it is further typical for the ankle joint in support phase to exhibit considerably increased plantar flexion; particularly increased are also knee flexion in the support phase and at the end of the swing phase as well as hip flexion throughout the gait cycle. Consequently, the ankle plantar flexion moment graph displays characteristic increase in early and midstance phase and decrease during push-off, in the knee moment graph we noticed considerably pronounced extension moment throughout the stance phase, whereas in the hip we recorded considerably increased extension moment in the early stance phase and increased flexion moment in the terminal stance phase. Although the child persisted in the toe walking gait pattern after inhibitory casting, the improvement is clearly evident. After inhibitory casting we noticed a significant shift of the ankle joint trajectory towards normal walking and noteworthy evolution of somewhat more extended posture in the knee as well as in the hip. This additionally led towards larger, almost normal ankle plantar flexion moment during push off, knee extension moment decreased but remained well above the normal level, whereas both hip extension and flexion moment bursts in early stance and terminal stance phases decreased, respectively.

### Simulation experiment

Clinical observations of toe walking dictated the selection of such a sequence of *ζ*_*i*_that encoded the following simulation objectives: when compared to normal walking *ζ*_1_ had to induce i) pronounced plantar flexion, ii) increased knee and hip flexion, iii) pronounced ankle plantar flexion moment in the early stance phase and somewhat decreased ankle plantar flexion moment during push-off, and iv) pronounced knee extension moment in the stance phase. After settling at the desired gait velocity, *ζ*_1_ (and successive *ζ*_*i*_) were modified according to (13) in such a way as to gradually encode the primary effects of inhibitory casting - releasing the tension in the plantar flexor muscle group - which considerably reduces kinematic constraint predominantly in the ankle joint. In terms of *ζ*_*i*_, such intervention was encoded predominantly by gradually decreasing *L*_*st*,*ankle*,*mid*_, *L*_*st*,*ankle*,*end*_, *L*_*sw*,*ankle*,*mid*_ and *L*_*sw*,*ankle*,*end*_ as well as by gradually increasing ${\dot{L}}_{\mathrm{st},\mathrm{ankle},\mathrm{end}}$ and ${\dot{L}}_{\mathrm{sw},\mathrm{ankle},\mathrm{end}}$. We expected that such progressive manipulation with the set of control parameters *ζ*_*i*_ (13) should gradually result in qualitatively similar kinematic and kinetic improvements as recorded in the selected clinical case (Figure 5): v) ankle plantar flexion should decrease, vi) knee and hip joints should allow more outstretched posture, vii) ankle plantar flexion moment during push-off should shift towards the normal pattern, and viii) knee extension moment should decrease.

To assure model stability any change in *ζ*_*i*_was performed manually after the model became stable at desired gait velocity and with a sufficiently subtle rate of change in *δζ*_*i*−1,*i*_. We used the standard deviation of gait velocity in the last *N* steps *σ*_*N*_(*v*_*gait*_) as a measure of gait stability. Gait kinematics and kinetics in the *k*-th step was considered stable if

(14) $$\sigma_{N}(v_{\mathrm{gait}})<\varepsilon$$

where *ε* denotes a sufficiently small level of permissible deviation from the desired gait velocity. Given the slow rate of change in *δζ*_*i*−1,*i*_ the simulation experiment spanned over more than five hundred successive gait cycles. We selected gait kinematics and kinetics in five gait cycles that best demonstrate the development of the desired gait characteristics. Their corresponding *ζ*_*i*_ are listed in Table 2.

**Table 2:** **Values of ***ζ*_i _**in selected gait cycles**

***ζ***		***π*_1_**	***π*_2_**	***π*_3_**	***π*_4_**	***π*_5_**			
*L*_*st*,*v*,*mid*_	cm	79.6	79.6	80.0	80.0	80.3			
*L*_*st*,*v*,*end*_	cm	82.0	82.0	83.6	81.1	81.9			
*q*_*P*,*mid*_	rad	-0.05	-0.05	-0.05	-0.05	-0.05			
*q*_*P*,*end*_	rad	-0.05	-0.05	-0.05	-0.05	-0.05			
${\dot{q}}_{P,\mathrm{end}}$	rad/s	-0.15	-0.15	-0.15	-0.15	-0.15			
*q*_*T*,*mid*_	rad	0	0	0	0	0			
*q*_*T*,*end*_	rad	0	0	0	0	0			
${\dot{q}}_{T,\mathrm{end}}$	rad/s	-0.15	-0.15	-0.15	-0.15	-0.15			
*L*_*st*,*ankle*,*mid*_	cm	**8.6**	**7.2**	**5.6**	**5.6**	**5.6**			
*L*_*st*,*ankle*,*end*_	cm	**9.4**	**8.6**	**6.2**	**6.2**	**6.9**			
${\dot{L}}_{\mathrm{st},\mathrm{ankle},\mathrm{end}}$	cm/s	**10**	**10**	**20**	**20**	**20**			
*L*_*sw*,*ankle*,*mid*_	cm	**8.4**	**8.4**	**8.1**	**8.1**	**8.1**			
*L*_*sw*,*ankle*,*end*_	cm	**7.6**	**7.6**	**6.2**	**6.8**	**6.8**			
${\dot{L}}_{\mathrm{sw},\mathrm{ankle},\mathrm{end}}$	cm/s	**0**	**0**	**10**	**10**	**10**			

### Data processing

It is common in biomechanics to present the gait pattern of an individual as a combination of gait kinematics and kinetics for one leg and for the whole gait cycle (i.e. between two consecutive contacts of the same leg). For this reason we gathered the simulation results for one side in two consecutive steps and assumed that a single support phase of one leg corresponds to a swing phase of the opposite leg. Additionally, kinetic data were filtered by using a fourth order Butterworth filter with cutoff frequency *f*_*c*_ = 5*Hz*.

## Results

**Figure 6:**
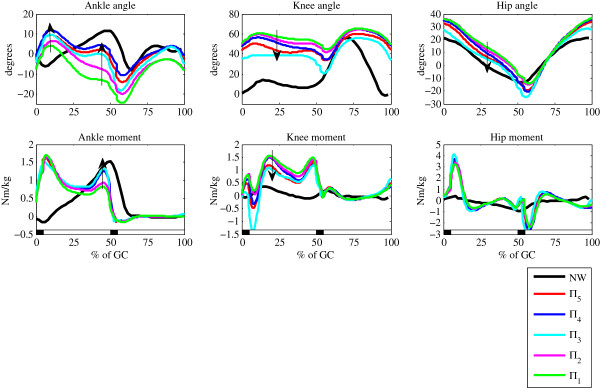
**Developing kinematic diversity in gait simulations by changing the set of control parameters ζ.** The sequence of black and white rectangles below the three graphs in the lower panel indicate consecutive phases of double support phase (0–5% of GC), left leg single support phase (5–50% of GC), double support phase (50–55% of GC) and left leg swing phase (55–100% of GC). Two transitions from black to white (5% of GC and 55% of GC) indicate right leg and left leg take-off respectively, whereas transition from white to black (50% of GC and 100% of GC) indicates the right leg and left leg contact phase respectively. Additionally, the interval 0–10% of GC is referred to as early stance phase, the interval 10–40% is referred to as midstance, the push off indicates the interval 40–50% of GC and the interval 50–55% denotes terminal stance phase.

Figure 6 shows simulation results in five selected gait cycles. When qualitatively inspecting kinematics and kinetics, we notice that all simulation cases developed the principal characteristics of toe walking. Moreover, by progressively changing the set of control parameters *ζ*_*i*_ as listed in Table 2, we were able to generate a sequence of toe walking gait kinematics and kinetics that gradually developed similar improvement tendencies (indicated by arrows) as recorded in the clinical case of toe walking after inhibitory casting (Figure 5). Typically pronounced ankle plantar flexion in the stance phase gradually decreased and shifted toward the normal range of ankle movement while retaining the toe walking pattern. In the knee joint approximately 60 degrees of initial knee flexion was reduced to less than 40 degrees after gradually changing the set of control parameters. A similar result is present in the hip joint - generally increased hip flexion throughout the stance and hip extension in terminal stance gradually shifted towards normal hip kinematics.

The ankle moment graph shows in all gait cycles a characteristic double teeth ankle plantar flexion moment that is generally present also in clinical cases of toe walking. After gradually changing the set of control parameters *ζ*_*i*_ the first peak in early and mid stance remained unchanged, whereas the second peak during push-off gradually increased from a typically modest ankle plantar flexion moment to almost normal range. In the knee joint, the initially pronounced knee extension moment diminished almost by half after changing the set of control parameters *ζ* whereas in the hip joint the effect of varying the set of control parameters *ζ* had a negligible effect - all simulation cases show large extension and flexion moment bursts in the early stance and early swing phase, respectively, that to some extent coincide with the increased extension moment in the early stance and increased flexion moment in the early swing phase.

In terms of selected *ζ* (Table 2) we notice that these changing trends in gait kinematics and kinetics correspond predominantly to gradual shortenings in the ankle components of virtual stance and swing leg. Decreasing *L*_*st*,*ankle*,*mid*_, *L*_*st*,*ankle*,*end*_, *L*_*sw*,*ankle*,*mid*_ and *L*_*sw*,*ankle*,*end*_ up to 35 % not only changed the ankle kinematics and kinetics; due to the relatively constant length of the virtual stance leg (*L*_*st*,*v*,*mid*_ and *L*_*st*,*v*,*end*_) they also imposed a more outstretched posture in the knee joint. Such improvements are typically present after undergoing inhibitory casting in CP children.

## Discussion

In the development of our toe walking model, our aim was to form a framework in which the joint movements would not need to be directly defined but could be inherently encoded in a set of general walking premises that are imposed through parameterized reference functions. The rationale behind imposition of the desired walking demands instead of following prerecorded joint angle trajectories is that in the latter approach each gait pattern requires its own set of joint angle references, and any deviation from the existing joint movements significantly aggravates model stability. On the other hand, in our simulation experiment we have shown that by encoding joint motions via a set of walking demands, the joint angles are free to develop so as to comply with these demands, and that changing only one walking demand in general imposes entirely new gait kinematics and kinetics on all joints without jeopardizing model stability. This closely relates to the typical situation in pathological gait treatment. Namely, in clinical gait analysis and treatment the primary effect of a certain anomaly or treatment is often known only locally, whereas the secondary compensatory changes which arise in response to a primary anomaly do change the gait kinematics and kinetics globally. For example, plantar flexor muscle contracture primarily increases ankle plantar flexion and reduces push-off. On the other hand, the human neural system may compensate for the lack of push-off by tilting the torso forward, increasing hip moment by recruiting hip muscles or placing an additional load on the knee joint, depending on the state of the muscles involved. It is a challenging task that requires a lot of experience to distinguish between primary anomalies and secondary compensations, as any deviation in gait kinematics and kinetics from the normal gait occurs due to the combined effect of primary as well as compensatory anomalies. It is also a crucial step in pathological gait treatment, as only primary anomalies should be treated, whereas secondary changes will disappear when they are no longer needed.

We envision the following situation in which generating new gait kinematics and kinetics by gradually changing the walking premises in a simulation model could complement the conventional approach to pathological gait treatment. When initially being acquainted with the patient’s gait kinematics and kinetics the therapist would reconstruct the patient’s gait kinematics and kinetics by systematically tuning walking premises in the simulation model until the gait kinematics and kinetics of the patient and the model were matched: in each iteration the therapist would gradually and by growing *δζ*_*i*−1,1_ change the set of normal walking premises as defined by the set of normal control parameters *ζ*_*normal*_ in such a way that the resulting *ζ*_*patient*_ would impose gait kinematics and kinetics that would correspond sufficiently well to the gait kinematics and kinetics of the patient. To simplify, let us assume that to achieve this it would suffice to gradually adjust the value of only the k-th parameter $\zeta_{i}^{k}$ in each iteration. The validity of such an assumption proceeds from the ability to modify gait kinematics and kinetics in all joints by adjusting the value of only one control parameter that in turn adjusts the corresponding walking premise. Once kinematics and kinetics of the patient and the simulation model were matched, a team of clinicians, therapists and engineers would interpret the discrepancies between $\zeta_{\mathrm{normal}}^{k}$ and $\zeta_{\mathrm{patient}}^{k}$ in terms of the most likely primary reason. Therefore, instead of establishing causal relationships between all deviations in gait kinematics and kinetics, the interpretation of gait kinematics and kinetics by means of simulation models would focus on determining the primary anomaly that is responsible for a particular discrepancy between $\zeta_{\mathrm{normal}}^{k}$ and $\zeta_{\mathrm{patient}}^{k}$. This would immediately grade some primary causes which they would usually consider as very unlikely and would significantly narrow the range of available therapeutical interventions, which would significantly facilitate the decision about the most promising treatment. Since the primary effects of most common interventions are well delineated and can be easily encoded as modification *δζ*_*therapy*_, the therapist would in subsequent treatment planning gradually change the existing walking premises *ζ*_*patient*,*new*_ = *ζ*_*patient*_ + *δζ*_*therapy*_. Hence, the model would predict new gait kinematics and kinetics which would reflect the effects due to the selected therapeutical intervention.

Due to the absence of *ζ*_*normal*_and structural simplicity of our toe walking model which aggravates reconstruction of the actual gait pattern, we were able only partly to illustrate this procedure in our simulation experiment. By following simulation objectives i-viii as defined in section Simulation experiment, we initially established such a set of control parameters *ζ*_*patient*_=*ζ*(*π*_1_) (Table 2) which induced toe walking gait kinematics and kinetics *π*_1_with similar toe walking characteristics as present in the selected clinical case of toe walking before the intervention (Figure 5) - simulation objectives i-iv. Even though *ζ*_*normal*_ was not available, *ζ*_*patient*_ (more precisely high *L*_*st*,*ankle*,*mid*_, *L*_*st*,*ankle*,*end*_, *L*_*sw*,*ankle*,*mid*_ and *L*_*sw*,*ankle*,*end*_) clearly indicates pronounced plantar flexion. Experience from clinical practice shows that inhibitory casting primarily decreases ankle plantar flexion and increases dynamics of the movement. To investigate the effect of such intervention in this particular clinical case of toe walking we encoded inhibitory casting by gradually reducing *L*_*st*,*ankle*,*mid*_, *L*_*st*,*ankle*,*end*_*L*_*st*,*ankle*,*mid*_ and *L*_*st*,*ankle*,*end*_ as well as increasing ${\dot{L}}_{\mathrm{st},\mathrm{ankle},\mathrm{end}}$ and ${\dot{L}}_{\mathrm{sw},\mathrm{ankle},\mathrm{end}}$ according to *ζ*(*π*_2_)…*ζ*(*π*_5_) in Table 2. Despite toe walking gait characteristics still being present, gait kinematics and kinetics *π*_2_…*π*_5_ (Figure 6) in general moved towards the normal range of movement and complied with the simulation objectives v-viii. Since the new gait pattern may be considered as less demanding, we may assume inhibitory casting to be an intervention with a potentially positive outcome. To evaluate the simulation results, they were compared to the actual clinical case of toe walking where the patient underwent inhibitory casting (Figure 5). By focusing on generally desired improvements (simulation objectives v-viii), rather than reproducing the clinical outcome, we notice that selected simulation gait cycles follow similar improvement tendencies (in Figure 6 they are marked with arrows) to those which one can identify in gait kinematics and kinetics after inhibitory casting in the selected clinical case (Figure 5).

The limitations of the proposed approach predominantly proceed from the simplified structure of the simulation model. The small number of degrees of freedom that confines the motion to only the sagittal plane significantly reduces the feasible range of movement of the model and cannot adequately account for compensations that often occur in transversal or coronal planes of motion. The main argument why focusing on sagittal plane only may be a reasonable compromise is that compensations are considerably better understood in transversal and coronal planes than in the sagittal plane, which is the plane of forward propulsion and progression. Nevertheless, expanding the feasible range of motion to the transversal (and coronal) plane of motion would improve the applicability of such a model, especially since human gait is synchronized and interrelated motion in all three planes of motion and should be treated as such. This is a precondition for successful and accurate reconstruction of patient specific gait kinematics and kinetics. At the current stage of toe walking model development, the reconstruction of human locomotion is feasible only to the extent that is supported by the modest structural complexity of the model. That is why reproduction of the patient’s gait kinematics and kinetics was beyond the scope of this research and is the subject of future work. Instead, we focused on construction of the principal characteristics of toe walking and qualitatively similar improvement tendencies to those which one can observe in the selected clinical case of toe walking. On the other hand, the desire to accurately reconstruct a particular gait pattern presumes negligible adjustments of the proposed control strategy. Namely, to extrapolate the feasible range of movement to all planes of motion, one would only need to extend the existing set of walking premises with additional walking premises and introduce them as new control parameters to the two-level control strategy without interfering with the overall control scheme (Figure 3). Such a model would then be suitable for more challenging clinical cases where the gait is changed due to the combined effect of more than one incorrect muscle functionalities.

Following the example of [24-26], the method as presented in this paper could also combine feedback control and static optimization techniques to calculate muscle forces. Thus the gait analysis could be extended to a muscle recruitment level instead of joint moments. We further suggest yet another optimization level which, in the process of gait diagnostics, would determine the set of control parameters in such a way as to minimize the deviations between the simulated gait pattern and the gait pattern of a particular patient, prerecorded by means of instrumented gait analysis. This would not only speed up the diagnostic process but would also optimize walking demands with respect to each individual. We also believe this would diminish the undesirable effects of the contact phase and switching control strategy. The rigid contact model assumes velocity discontinuities when switching from single to double support, whereas the switching control strategy imposes two large hip moment bursts at the beginning of the single support and the beginning of the swing phase (the opposite leg is in single support), respectively. While they are consistent with clinical observations of toe walking (Figure 5) it is very likely that the majority of surplus arises due to rapid correction of asymmetry from the end of double support. This directly affects the knee moment as well. To meet control demands, the knee actuator must simultaneously provide sufficient knee flexion moment to compensate for excessive hip extension moment, which would otherwise force the knee joint to extend.

## Conclusions

Simulation experiments have shown that the proposed framework is capable of generating primary toe walking characteristics as recorded in a selected clinical case of toe walking. Also, when adjusting the set of control parameters so as to encode the primary effect of inhibitory casting, which is a typical therapeutical intervention of toe walking, the resulting gait kinematics and kinetics follow similar improvement tendencies to those which one can identify in gait kinematics and kinetics after inhibitory casting in the selected clinical case. This suggests the potentially practical implication of the proposed framework in clinical gait assessment and therapy planning in the future.

## Competing interests

The authors declare that they have no competing interests.

## Author’s contributions

Both authors significantly contributed to the conception, theoretical analysis, simulation experiments, data interpretation and writing of the manuscript. Both authors revised and approved the final manuscript.
